# Evolution of changes in cognitive function after the initiation of antiretroviral therapy

**DOI:** 10.1186/s12981-016-0104-0

**Published:** 2016-04-14

**Authors:** Borja Mora-Peris, Elizabeth Stevens, Francesca Ferretti, Jonathan Underwood, Stephen Taylor, Alan Winston

**Affiliations:** Clinical Trials, Faculty of Medicine, Winston Churchill Wing, St. Mary’s Hospital, Imperial College, Praed Street, London, W2 1NY UK; Department of HIV and Genitourinary Medicine, Imperial College Healthcare NHS Trust St Mary’s Hospital, London, UK; Birmingham Heartlands HIV Service, Heart of England NHS Foundation Trust, Birmingham, UK; Department of Infectious Diseases, San Raffaele Scientific Institute, Milan, Italy; Division of Immunity and Infection, University of Birmingham, Birmingham, UK

**Keywords:** Cognitive function, cART, Naïve, HIV, Nevirapine

## Abstract

**Background:**

Cognitive function is reported to improve after the initiation of combination antiretroviral therapy (cART). Data on the evolution of such changes are limited. We assessed the dynamics of changes in cognitive parameters, in HIV-positive subjects initiating cART.

**Methods:**

Cognitive function in seven domains was evaluated for HIV-infected patients without clinically significant cognitive impairment prior to the initiation of cART, and 24 and 48 weeks after. Cognitive scores were transformed using standardised z-scores according to the pooled baseline standard deviation. Global, speed, and accuracy composite z-scores were calculated with changes calculated using a paired t test.

**Results:**

In 14 subjects, change in global cognitive z-scores from baseline was by 0.08 at week 24 (p = 0.59) and 0.15 at week 48 (p = 0.43). Change in composite speed and accuracy *z*-scores from baseline at weeks 24/48 were 0.07/0.05 (p = 0.45/0.82) and 0.13/0.23 (p = 0.47/0.45), respectively. In two of the cognitive domains assessing speed (learning and monitoring time), a continued improvement from baseline to weeks 24 and 48 was observed (changes of 0.06–0.08 and 0.10–0.19, respectively), whereas in two domains (detection and identification) an initial improvement at week 24 (changes of −0.10 and 0.04 from baseline, respectively) was followed by a deterioration in score at week 48 (changes of −0.12 and −0.08 from baseline, respectively). None of these changes were statistically significant.

**Conclusions:**

A trend for improvement in cognitive function was observed in naïve HIV-positive patients starting cART. The dynamics of this improvement differed both between cognitive domains and the time-points assessed.

## Background

Cognitive impairment is a frequently reported complication in otherwise effectively treated HIV-positive individuals [1, 2]. The presence of HIV-associated cognitive impairment has been associated with reduced quality of life and higher mortality [3, 4].

In general, improvements in cognitive function have been reported in patients initiating antiretroviral therapy [5–7]. Data describing the dynamics of changes in cognitive function after the initiation of cART are sparse [8, 9]. An understanding of the dynamics of these changes is crucial to assist in the design of future research programmes assessing cognitive function within longitudinal cohorts and to assist in diagnostic algorithms for cognitive impairment in treated HIV-disease.

The aim of this study was to assess changes in cognitive parameters and the dynamics of these changes within individual cognitive domains in people living with HIV (PLWH) commencing antiretroviral therapy.

## Methods

### Study design and procedures

This analysis was undertaken as part of an open-labelled, randomised, prospective clinical trial conducted at Imperial College Healthcare NHS Trust (St Mary’s Hospital, London, UK) and the Heart of England NHS Foundation Trust (Birmingham, UK) between February 2008 and October 2012.

Within the study protocol, subjects were randomised on a 1:1 basis to emtricitabine (200 mg once daily) + tenofovir disoproxil fumarate (245 mg once daily) plus either atazanavir (300 mg once daily) + ritonavir (100 mg once daily) or nevirapine 400 mg (once daily).

### Subject selection

ART-naïve, HIV-positive patients aged over 18, with no clinically overt cognitive impairment or abnormalities on screening laboratory testing were eligible to participate. In line with national prescribing guidelines for the use of nevirapine as an antiretroviral agent, CD4+ lymphocyte count <400 cells/μL and <250 cells/μL were required for males and females, respectively. Exclusion criteria included current history of major psychiatric disorders, significant co-morbidities, use of concomitant medication with potential drug–drug interactions, or evidence of HIV-1 genotypic resistance mutations.

This study was registered in the European Clinical Trials Database (EudraCT number 2007-002405-47) and national human ethics committee approval was granted prior to recruitment. All patients gave informed consent before screening.

### Cognitive testing

A computerised cognitive test battery (CogState™) [10, 11] was performed at baseline, weeks 24 and 48. This battery comprised of seven tasks and required approximately 10–15 min to complete. The tasks were in the form of card games and specifically assessed the following domains: detection, identification, monitoring and matched learning (all assessed via speed of test); associate learning, one card learning and working memory (assessed via accuracy of test). All study participants completed one full practise test prior to study examination to optimize performance at baseline.

### Statistical methods

We initially aimed to recruit 40. Given only 14 subjects completed our study, this analysis is descriptive with no formal comparisons across treatment arms..

All statistical calculations were performed using SPSS (version 22.0; SPSS Inc., Chicago, IL, USA) and analysis was conducted according to CogState™ recommendations. Reaction times were log_10_-transformed because of a positive skew of the distribution, and accuracy measures were transformed using arcsine-root transformation. For each subject, results were standardized as z-scores according to the pooled baseline standard deviation (SD) for each domain.

Composite z-scores were calculated overall (global) and for the speed and accuracy domains based on the average of standardized z-scores. Changes from baseline scores to weeks 24 and 48 were calculated for each individual and composite domain. For the calculation of composite scores and changes, z-scores involving speed domains were multiplied by −1. This transformation allowed for all parameters a positive change to represent an improvement in function whereas a negative change from baseline represented deterioration in function. Statistical significance of changes in scores from baseline to weeks 24 and 48 was assessed for every parameter accordingly using a paired t test.

Only validated cognitive results were incorporated into the analysis.

## Results

### Patient characteristics

24 subjects were screened, 20 were enrolled and 14 completed all study procedures. Among those six who did not complete study procedures, four dropped out and two lacked complete cognitive data. Patient characteristics are summarised in Table 1. Plasma HIV RNA was <50 copies/mL at weeks 36 and 48 (Table 1) in all subjects.

**Table 1:** Patient demographics and clinical characteristics throughout the study

Parameter^a^	(n=14)
Age (years; range)	37.4 (23–48)
Male, n (%)	14 (100 %)
Ethnicity (%)	
White	9 (64.3 %)
Black	3 (21.4 %)
Asian	2 (14.3 %)
Baseline CD4+ count (cells/μL)	280 (129)
Baseline HIV RNA (log copies/μL)	4.42 (0.58)
HIV-1 clade B, n (%)	10 (71.4 %)
Patients with HIV RNA <50 copies/μL, n (%)	
Week 24	11 (78.6 %)
Week 36	14 (100 %)
Week 48	14 (100 %)

^a^Mean (SD) unless otherwise stated. No statistically significant differences (p > 0.1) for any parameters between study arms

### Cognitive function changes over 48 weeks

Overall, there were improvements in cognitive function over the study period but these changes were not statistically significant. For instance, global composite changes in z-scores (SD) increased from 0.08 (0.54) at week 24 (p = 0.59) to 0.15 (0.60) at week 48 (p = 0.43) (Table 2). Likewise, the changes from baseline in composite accuracy speed *z*-scores (SD) increased from 0.13 (0.63) at week 24 to 0.23 (0.93) at week 48. Such improvements in z-scores were also observed in the composite speed *z*-scores: 0.07 (0.37) at week 24 and remained stable by 0.05 (0.67) at week 48.

**Table 2:** Changes in Z-score neurocognitive testing parameters at weeks 24 and 48 from baseline

Cognitive domain	Overall			
	*n*	Z-score change	SD of change	p value
Detection^a^				
W24	14	0.10	0.68	0.59
W48	12	−0.12	0.79	0.61
Identification^a^				
W24	14	0.04	0.75	0.84
W48	11	−0.08	0.99	0.78
Matched learning^a^				
W24	14	0.06	0.71	0.76
W48	11	0.08	0.99	0.78
Monitoring time^a^				
W24	14	0.10	0.64	0.55
W48	11	0.19	0.78	0.43
Associate learning^b^				
W24	13	0.01	0.08	0.65
W48	10	0.19	0.64	0.36
One card learning^b^				
W24	14	0.17	1.27	0.62
W48	12	0.11	1.46	0.78
Working memory^b^				
W24	14	0.41	1.00	0.14
W48	11	0.34	1.30	0.41
Composite speed^a^				
W24	14	0.07	0.37	0.45
W48	11	0.05	0.67	0.82
Composite accuracy^b^				
W24	13	0.13	0.63	0.47
W48	10	0.23	0.93	0.45
Global composite^c^				
W24	13	0.08	0.54	0.59
W48	10	0.15	0.60	0.43

All change estimates are based on pooled-standard-deviation standardized z-score changes

*N* number of subjects, *SD* standard deviation of the change, *CI* confidence interval. Shaded areas, z-score changes

^a^Used to determine speed (sp); a positive change in z-score represents an improved response

^b^Used to determine accuracy (acc) of response; a positive change in z-score represents an improved response

^c^A positive change in z-score represents an improved response. Global composite = composite accuracy + speed

All three accuracy domains (associate learning, one card learning and working memory) showed an improvement from baseline to week 24 (z-score changes of 0.01, 0.17 and 0.41, respectively). At week 48, such improvements were sustained for one card learning and working memory (z-score changes of 0.11 and 0.34 from baseline, respectively) and increased for associate learning (z-score change of 0.19 from baseline).

Speed domains such as matched learning and monitoring time showed a continued improvement from baseline to week 24 and then to week 48 (changes of 0.06 to 0.08 and 0.10 to 0.19, respectively), whereas others such as detection and identification showed an initial improvement at week 24 (changes of 0.10 and 0.04 from baseline, respectively) followed by a small decline at week 48 (changes of −0.12 and −0.08 from baseline, respectively) (Table 2).

## Discussion

We assessed changes in cognitive parameters in HIV-positive subjects at 24 and 48 weeks after commencing cART for the first time.

We observed general trends suggesting an initial improvement in composite speed and accuracy cognitive domains at week 24, which continued to week 48 after starting cART. However, none of these trends were of statistical significance, which may be due to the small number of subjects studied.

Previous studies have reported overall neuropsychological function improvement to peak around 24–36 weeks after cART initiation with these improvements prolonged up to week 48 [6]. Similarly, in the ALTAIR study speed and accuracy domains improved over the first year after cART initiation with no individual domain showing deterioration from baseline at week 48 [7]. However, in the long term follow up of this study a decline in global cognitive function score was observed over 144 weeks and the authors hypothesised this could be related to antiretroviral toxicities [12]. In our study, domains critically affected in HIV-associated cognitive impairment such as detection and identification times [13] deteriorated slightly at week 48 after an initial improvement at week 24 (Table 2). This highlights that different dynamics of change are observed in different cognitive domains including potential fluctuations over time suggesting that different domains should be explored independently as well as a part of global cognitive assessments. Some limitations to consider while interpreting these data are the likelihood of a learning effect despite a practice neurocognitive test at screening and the open-label nature of the study. Additionally, the lack of clinically significant cognitive impairment in the study population may have reduced the power to detect significant improvements. We recruited subjects without overt cognitive impairment based on clinical judgement. Given we do not have an appropriate control dataset we are unable to classify subjects into cognitively impaired and non-impaired based on the cognitive testing results in our study, which would be of interest after the initiation of cART [8].

We had initially planned to undertake a comparative study of two different antiretroviral treatment regimens. However, recruitment was challenging. We believe the use of nevirapine was one of the factors underlying these recruitment difficulties. Firstly, nevirapine was not recommended as preferred first line treatment for naïve patients in the UK by the time the study commenced in 2008 [14]. Secondly, only patients with low CD4 counts (<400 cells/μL and <250 cells/μL for males and females, respectively) were eligible to enter this study due to the reported increased risk of toxicity for patients with higher CD4 counts commencing nevirapine-containing cART regimens [14]. This limited the eligible population for our study. The HIV-therapeutic field continues to be a rapidly changing arena. Recent guidelines changes include recommending treatment for all PLWH, irrespective of CD4+ lymphocyte count, in view of the results from the INSIGHT-START study [15]. Therefore studies stipulating CD4+ count criteria at entry are no longer feasible or ethical. Recent treatment guidelines are swaying towards commencing therapy with an integrase-inhibitor containing regimen [16, 17]. Such changes make recruitment to studies using regimens containing non-nucleoside reverse transcriptase inhibitors such as efavirenz, which have been standard of care for almost a decade, challenging.

In summary, a general trend of improvement in cognitive function parameters was observed in naïve HIV-positive participants initiating antiretroviral therapy with the dynamics of these changes differing between cognitive parameters over 48 weeks of follow-up.
